# A Novel Method for Assessment of Natural Killer Cell Cytotoxicity Using Image Cytometry

**DOI:** 10.1371/journal.pone.0141074

**Published:** 2015-10-22

**Authors:** Srinivas S. Somanchi, Kelsey J. McCulley, Anitha Somanchi, Leo L. Chan, Dean A. Lee

**Affiliations:** 1 Division of Pediatrics, Cell Therapy Section, The University of Texas MD Anderson Cancer Center, Houston, Texas, 77030, United States of America; 2 Department of Technology R&D, Nexcelom Bioscience LLC, 360 Merrimack St. Building 9, Lawrence, Massachusetts, 01843, United States of America; 3 Graduate School of Biomedical Sciences, The University of Texas Health Sciences Center, Houston, Texas, 77030, United States of America; Harvard Medical School, UNITED STATES

## Abstract

Natural killer (NK) cells belong to the innate arm of the immune system and though activated NK cells can modulate immune responses through the secretion of cytokines, their primary effector function is through target cell lysis. Accordingly, cytotoxicity assays are central to studying NK cell function. The ^51^Chromium release assay, is the “gold standard” for cytotoxicity assay, however, due to concerns over toxicity associated with the use and disposal of radioactive compounds there is a significant interest in non-radioactive methods. We have previously used the calcein release assay as a non-radioactive alternative for studying NK cell cytotoxicity. In this study, we show that the calcein release assay varies in its dynamic range for different tumor targets, and that the entrapped calcein could remain unreleased within apoptotic bodies of lysed tumor targets or incompletely released resulting in underestimation of percent specific lysis. To overcome these limitations, we developed a novel cytotoxicity assay using the Cellometer Vision Image Cytometer and compared this method to standard calcein release assay for measuring NK cell cytotoxicity. Using tumor lines K562, 721.221, and Jurkat, we demonstrate here that image cytometry shows significantly higher percent specific lysis of the target cells compared to the standard calcein release assay within the same experimental setup. Image cytometry is able to accurately analyze live target cells by excluding dimmer cells and smaller apoptotic bodies from viable target cell counts. The image cytometry-based cytotoxicity assay is a simple, direct and sensitive method and is an appealing option for routine cytotoxicity assay.

## Introduction

Natural killer (NK) cells are innate immune cells that act as the first line of defense against tumor cells and various pathogens [1]. The effector functions of NK cells include immune regulation through secretion of cytokines such as interferon-γ and TNF-α by a minor subset (CD56^bright^ CD16^−^) [2]. However, the primary mode of action by the major subset of NK cells (CD56^dim^CD16^+^) is the direct lysis of their targets [3]. Therefore, assessment of NK cell cytolytic function is fundamental to the study of NK cell biology and application in adoptive immunotherapy.

The cytolytic activity of NK cells is assessed either through a degranulation assay (LAMP1/CD107a) [4] or through a cytotoxicity assay. The degranulation assay, although very useful in assessing percentage of NK cells that respond to a stimuli (such as a tumor target), it does not provide any information about the outcome of the response, such as cytolysis of the tumor targets following the degranulation assault by NK cells. Therefore cytotoxicity assays are important in the context of understanding the cytolytic impact of NK cells and to measure the sensitivity of a given tumor target for lysis by NK cells. Cytotoxicity assays are thus more commonly used to assess the functional efficacy of NK cells for adoptive immunotherapy applications. Several assays have been developed for determining cytotoxicity of immune cells; use of ^14^Chromium was first reported in 1964 [5] and the ^51^Chromium release assay (CRA) was described in 1968 [6]. To date, CRA is considered the ‘gold standard’ for measuring NK cell and cytolytic T cell cytotoxicity [7–11]. However, due to concerns over the toxicity of handling and disposing radioactive compounds several methods have been developed as alternatives to CRA. One alternative method based on a non-toxic fluorescent dye using Calcein AM (acetoxymethyl) was developed in 1994 [12]. Other methods include flow-based cytotoxicity assays [13–17], LDH release assays [18–20], and more recently, a bioluminescence-based method [21]. Some of these methods show good correlation of target cell lysis to CRA [17, 22, 23], while others show greater target cell lysis than CRA [13, 21]. The calcein release assay was shown by Neri S. *et al*. to have good correlation to CRA at assessing percent specific lysis [23]. Hence we have routinely used the calcein release assay for reporting NK cell cytotoxicity in our studies [24, 25].

However, we have observed that calcein has a divergent loading efficiency in different cell lines and calcein has been shown to have higher spontaneous release compared to ^51^Chromium (^51^Cr) [23]. High spontaneous release and lower loading efficiency in some tumor cell lines could lead to reduced dynamic range and diminished sensitivity of the assay. Additionally, as the calcein release assays measure target cell lysis by the release of entrapped calcein into the supernatant, an incomplete release of calcein from lysed cells could result in underestimation of the percent lysis of target cells. Target cell death following interaction with immune effectors such as NK cells and cytotoxic T lymphocytes is driven either through perforin mediated osmotic lysis / necrotic death [26], or through an apoptotic pathway mediated by granzyme B and death receptors such as TNF-related apoptosis inducing ligand (TRAIL) and Fas ligand [27, 28]. While necrotic lysis of the targets could lead to emptying of the cellular contents into the culture supernatant through membrane damage, apoptotic death leads to formation of cellular ‘blebs’ or apoptotic bodies that could potentially retain cellular contents without releasing them into the supernatant. Apoptotic bodies could therefore prevent complete release of calcein from target cells, leading to underestimation of percent specific lysis by the calcein release assay.

Using live imaging of the calcein release assay we have demonstrated in this study that calcein loaded into target cells could in fact be retained within the apoptotic bodies following lysis by NK cells. To the best of our knowledge this is the first report to show retention of calcein within apoptotic bodies. Here we are reporting the development of a novel cytotoxicity assay using the Cellometer Vision Image Cytometer to overcome the limitations of variable loading, high spontaneous release, and incomplete release associated with the standard calcein release assay. Cellometer Vision CBA is an image cytometer that has been demonstrated to perform rapid and accurate cell counting, morphology analysis, and cell-based assays [29, 30]. In this study, we compared the efficiency of the image cytometry method to the standard calcein release assay previously reported [24] and demonstrate that the image cytometry method shows significantly higher percent specific lysis of the tumor targets compared to standard calcein release assay. We thus propose Image Cytometry as a simple and sensitive method for assessment of NK cell cytotoxicity with improved accuracy over standard calcein release assay.

## Materials and Methods

### Cells and Cell lines

The human tumor cell lines K562 (human chronic myelogenous leukemia, CML) and Jurkat cell lines (acute T leukemia) were obtained from ATCC, the 721.221 parental cell line (EBV transfected B cell line) was a gift from Dr. Laurence J.N. Cooper (MD Anderson Cancer Center) and MOLM 13 was a gift from Dr. Patrick Zweidler-McKay (MD Anderson Cancer Center). These cell lines were cultured in RPMI 1640 media (Cellgro/Mediatech, Manassas, VA) supplemented with 10% FBS (Life Technologies, Carlsbad, CA), 1x GlutaMAX (Gibco/Life Technologies, Carlsbad, CA) and 1x Pen/Strep (Cellgro/Mediatech, Manassas, VA), at 37°C in a 5% CO_2_ incubator.

The neuroblastoma cell lines, SK-N-BE(2), CHLA 155, CHP134 were generously provided by Dr. Patrick C. Reynolds (Texas Tech University Healthy Sciences Center), and IMR32 was procured from ATCC. These cells were cultured in complete IMDM media (Cellgro/Mediatech, Manassas, VA) supplemented with 10% FBS (Life Technologies, Carlsbad, CA), 1x GlutaMAX (Gibco/Life Technologies, Carlsbad, CA) and 1x Pen/Strep (Cellgro/Mediatech, Manassas, VA), at 37°C in a 5% CO_2_ incubator.

Buffy coat from 5 healthy donors was procured from the Gulf Coast Regional Blood Center under IRB-approved protocol (The University of Texas MD Anderson Cancer Center). The NK cells were expanded from peripheral blood mononuclear cells as previously described [24]. In brief, peripheral blood mononuclear cells were isolated from the buffy coat on Ficoll-Paque Plus (GE HealthCare, Piscataway, NJ) and were stimulated weekly for three weeks by co-culturing with irradiated (100Gy) K562 Clone9.mbIL21 in RPMI 1640 complete media (as above) supplemented with 50 IU/ml IL2 (Proleukin, Novartis Vaccines and Diagnostics, Inc). Half of the media was replaced every two days and supplemented with fresh IL2. The expanded NK cells were frozen after three weeks of expansion. For experiments using unexpanded primary NK cells, PBMCs were first isolated by Ficoll-Paque density centrifugation from buffy coat of healthy blood bank donors followed by negative selection of NK cells using RosetteSep^TM^ Human NK cell enrichment cocktail as previously described [24].

### Cytotoxicity Assay

The target tumor cells were stained with calcein for determining cytotoxic potential of NK Cells by the calcein release assay and Cellometer image cytometry. For staining target cells, a 2 μg/ml Calcein AM (Life Technologies, Carlsbad, CA) staining media was prepared (1 mg/ml stock) in complete RPMI media. The tumor cells were resuspended at 1 x 10^6^ cells/ml in Calcein AM-staining media and incubated for 30 minutes at 37°C in 5% CO_2_ incubator with intermittent mixing. The target cells were washed, and resuspended at 1 x 10^6^ cells/ml in RPMI complete media. Expanded NK cells were resuspended at 2 x 10^6^ cells/ml in RPMI complete media, and three serial dilutions (2 fold) was performed. Aliquots of 100 μl from each NK cell serial dilution containing 2 x 10^5^, 1 x 10^5^ and 0.5 x 10^5^ cells were added per well in a 96 well U bottom plate, in triplicates. Aliquots of 100 μl of Calcein loaded tumor cells were added (1 x 10^5^ cells/well) to each of these wells to generate 2:1, 1:1 and 0.5:1 effector—to-target ratio (E:T ratio). Maximum and spontaneous release controls were set up in 6 replicates using 1% Triton X-100 (final concentration) and plain media respectively. The plate was spun at 100 x g for 2 minutes and incubated for 4 hours at 37°C in 5% CO_2_ incubator. After the 4 hour incubation, the cells were gently mixed to evenly distribute the released calcein in the supernatant and the plate was spun at 400 x g for 2 minutes to pellet the cells and any debris.

For the calcein release assay, 150 μl of the supernatant was recovered and transferred to a flat bottom plate. The fluorescence was read using a BioTek Synergy 2 plate reader (Ex: 485 nm / Em: 530 nm). The percent specific lysis was calculated using the formula [(Test release−Spontaneous release)/(Maximum release−Spontaneous release)] x 100.

For the image cytometry assay, the cell pellet was gently and thoroughly resuspended in the remaining 50 μl of media and 20 μl was transferred to the Nexcelom disposable counting slide. Bright-field and fluorescence images of the sample were captured using the filter optics module VB-535-402 (Ex: 470 nm / Em: 535 nm) for calcein detection at an exposure time of 40 ms with a size cutoff of 0.1μm. The images were analyzed using FCS Express 4 (De Novo Software, Los Angeles, CA). The percent specific lysis was calculated from each sample using the formula [(Live fluorescent cell count in spontaneous−Live fluorescent cell count in test)/(Live fluorescent cell count in spontaneous)] x 100.

Additionally, calcein release assay and image cytometry methods were compared for assessing percent specific lysis of primary NK cells. The primary NK cells were resuspended at 2.5x10^6^/ml, and serially diluted (2 fold dilution) in triplicates in “U” bottom 96 well plate as before. Leukemia cell line MOLM-13 was stained with calcein (1:500 dilution), and resuspended at 2.5x10^5^ cells/ml. 100μl (25,000 cells) of MOLM-13 cells were added to each well containing NK cells generating E:T ratios ranging from 10:1 through 0.6:1. Six replicates of maximum release and spontaneous release controls each were included as before.

Live imaging of NK cell cytotoxicity was performed using Nikon Biostation IM-Q (Nikon Instruments, Inc) with two tumor targets. The neuroblastoma cell line CHP134 (loaded with calcein as before) was seeded in the Hi-Q4 (4 chambered) culture dish and allowed to adhere, then NK cells were added at an E:T ratio of 10:1 and the dish was imaged at predetermined locations. K562 tumor targets (loaded with calcein) and NK cells were seeded together in Hi-Q4 dish at an E:T ratio of 10:1. The images were acquired every 4–6 minutes.

To evaluate the dynamic range of calcein release assay, fluorescence data from maximum and spontaneous release controls from various tumor targets (K562, SK-N-BE(2), CHP134, CHLA155, IMR32, Jurkat, 721.221 and Nalm 6) loaded with calcein were quantitated and plotted after normalizing the maximum release data to 100%.

### Analysis and Statistics

For this comparative study on NK cell cytotoxicity assay using the calcein release assay and image cytometry, NK cells derived from 5 donors (effector cells n = 5) and three tumor targets (targets cells n = 3) were used. Each cytotoxicity assay was performed in triplicate. The fluorescence intensity data from image cytometry was analyzed using FCS Express 4 (De Novo Software), and the percent specific lysis was calculated based on the reported fluorescent live cell numbers. The average percent specific lysis for the 5 NK cell donors from the calcein release assay and image cytometry assay were analyzed and the statistical significance was calculated using Wilcoxon matched-pairs signed rank, non-parametric, two-tailed t test (Prism 6; Version 6.0e) and p < 0.05 was considered significant.

## Results

### Caveats of Standard Calcein Release Assay

**Fig 1:**
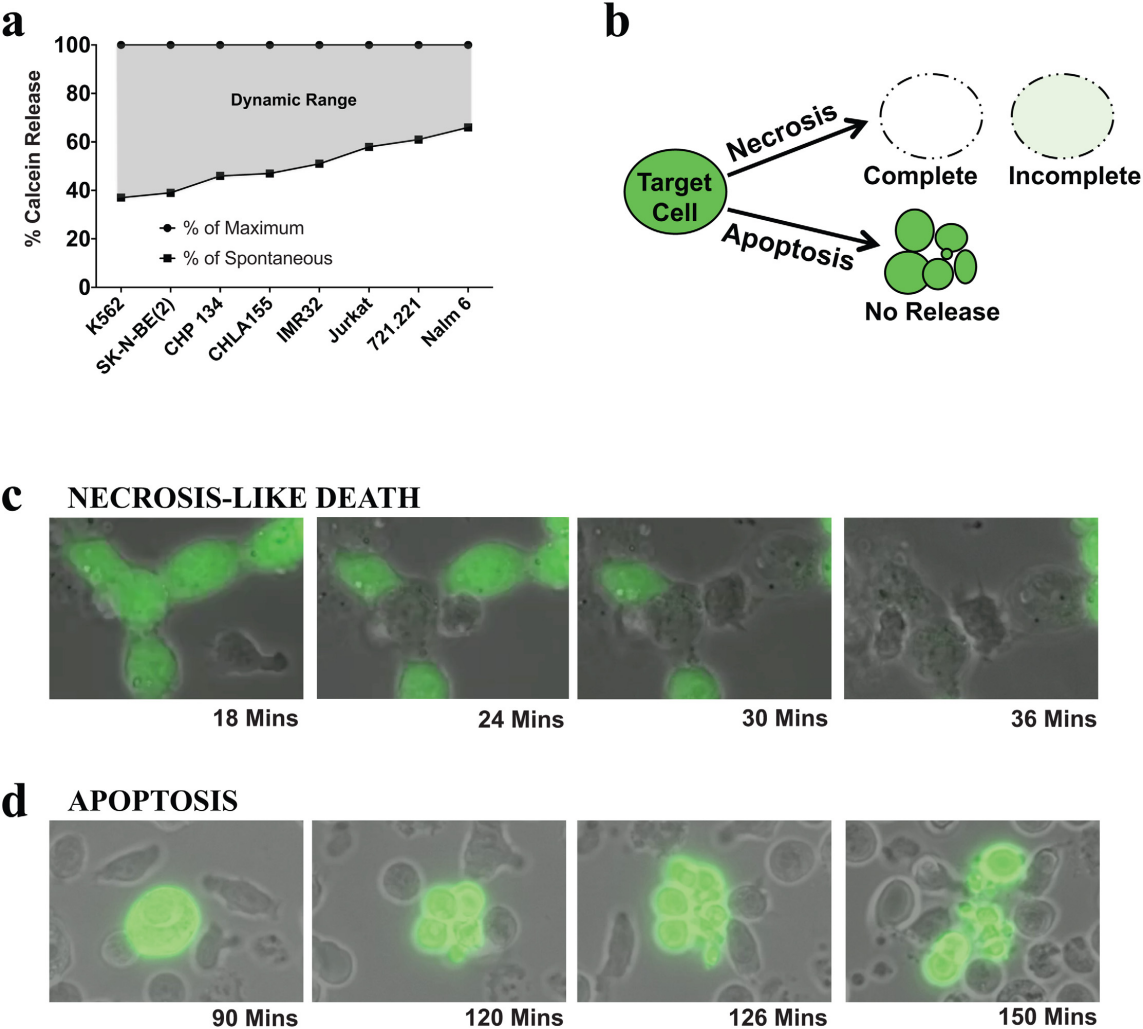
Sensitivity of calcein release assay. (a) Dynamic range for the calcein release assay was determined for neuroblastoma and leukemia tumor targets (K562, SK-N-BE(2), CHP134, CHLA155, IMR32, Jurkat, 721.221 and Nalm 6). The cell lines were stained with Calcein AM, and seeded in 96 well plates to generate maximum release (lysis with 1% TritonX-100) and spontaneous release data. After 4 hours the supernatant fluorescence was measured. The maximum release was normalized to 100% and the spontaneous release is represented as % of maximum. (b) Illustration of calcein release from target cells upon lysis by NK cells following necrosis-like and apoptotic death. (c) Bright-field and fluorescence overlay images of calcein release from CHP 134 cells undergoing necrosis-like death following interaction with NK cells. (d) Bright-field and fluorescent overlay images of calcein release from some K562 cells undergoing apoptotic death following interaction with NK cells. The images were derived from live Nikon Biostation IQ-M videos.

We have previously reported cytotoxicity of expanded NK cells against numerous tumor targets using the calcein release assay [24, 25]. Although this assay is easy to set up and avoids the use of toxic radioactive material, the calcein release assay has been shown to have high spontaneous release [23], additionally we have observed that the amount of calcein loading into cells is highly varied which together could reduce the dynamic range and thus sensitivity of the assay. Here, using the maximum and spontaneous release data for various cell lines, we have shown that the dynamic range available for the assay to measure percent specific lysis is highly varied between cell lines (Fig 1a), with K562 having the highest dynamic range amongst the cell lines shown.

Although not previously reported, it is conceivable that a portion of the calcein loaded into the target cells could be retained within target cell debris upon NK cell mediated necrosis-like cell death or substantially within the apoptotic bodies generated upon apoptotic lysis of the target cells as illustrated in Fig 1b.

Using live imaging of NK cell-mediated cytotoxicity, we have demonstrated here that an incomplete release of calcein from lysed cells and a substantial retention of calcein within apoptotic bodies does occur during cytolysis by NK cells. S1 Movie shows NK cell lysis of neuroblastoma tumor targets CHP134. The CHP 134 cells undergo rapid lysis releasing calcein in a necrosis-like cell death and in this case most of the calcein is released into the supernatant while a minute quantity remains entrapped evidenced by the green fluorescent hue in the cell debris. Fig 1c shows sequential images of CHP134 lysis by NK cells from the live cell imaging. S2 Movie illustrates apoptotic death noted in some K562 cells. During lysis by NK cells, some of these cells break up into apoptotic bodies. During such apoptotic death the calcein is retained within the apoptotic bodies and is not released into the supernatant. Sequential images of the apoptotic death of K562 are shown in Fig 1d.

Therefore, the narrow dynamic range of calcein release assay along with the retention of calcein within lysed targets or apoptotic bodies could significantly lower the percent specific lysis assayed by the calcein release assay, especially when compared to the complete lysis (solubilization) of target cells achieved by Triton X-100 in the Maximum release control.

### Image Cytometry for determining NK Cell cytotoxicity

**Fig 2:**
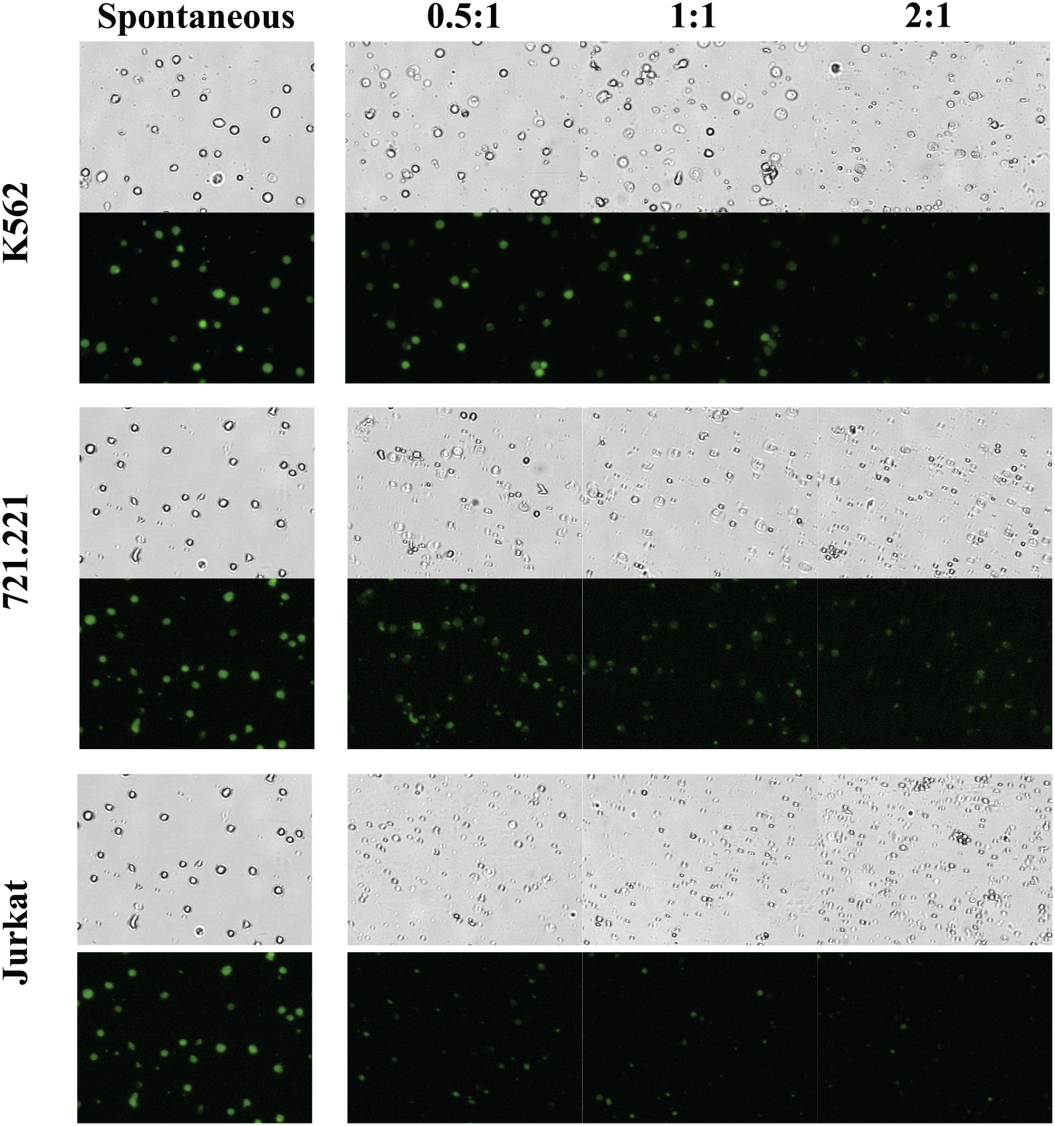
Assessment of NK cell cytotoxicity by image cytometry. Fluorescent images (from Image cytometer) of K562; 721.221 and Jurkat cell lines showing progressive loss of fluorescent (live) cells from the culture with increasing E:T ratio. The images from spontaneous serve as no treatment control. Representative images of target cell killing from one NK cell donor is shown for each cell line and E:T ratio.

To assess NK cell cytotoxicity by image cytometry we performed the cytotoxicity assay in a 96 well “U” bottom plate with 100,000 calcein loaded target cells per well and NK cells at an E:T ratio of 2:1, 1:1 and 0.5:1 in a total volume of 200 μl /well. The assay was performed using three tumor target cell lines: K562, 721.221 and Jurkat. At the end of 4-hour cytotoxicity assay, 150 μl of the supernatant was recovered and assessed by the standard calcein release assay for released calcein using a fluorescence spectrophotometer. The cell pellet was resuspended in the remaining 50 μl, and 20 μl of the cells were transferred to a disposable slide and imaged using the image cytometer. To determine percent specific lysis, bright-field and fluorescent images were captured to identify calcein loaded live target cells at each E:T ratio (in triplicates) and in spontaneous controls. Fig 2 shows representative bright-field and fluorescent images at each E:T ratio for the target cell lines. Target cells in the spontaneous control (without NK cells) exhibited brightly fluorescent live cells, while fewer fluorescent live target cells are present with increasing NK cell numbers (E:T ratio). At an E:T ratio of 2:1, almost a complete lysis of K562 cells was observed. 721.221 cells had nearly complete lysis at an E:T ratio of 1:1 and Jurkat cells at E:T ratio of 0.5:1.

**Fig 3:**
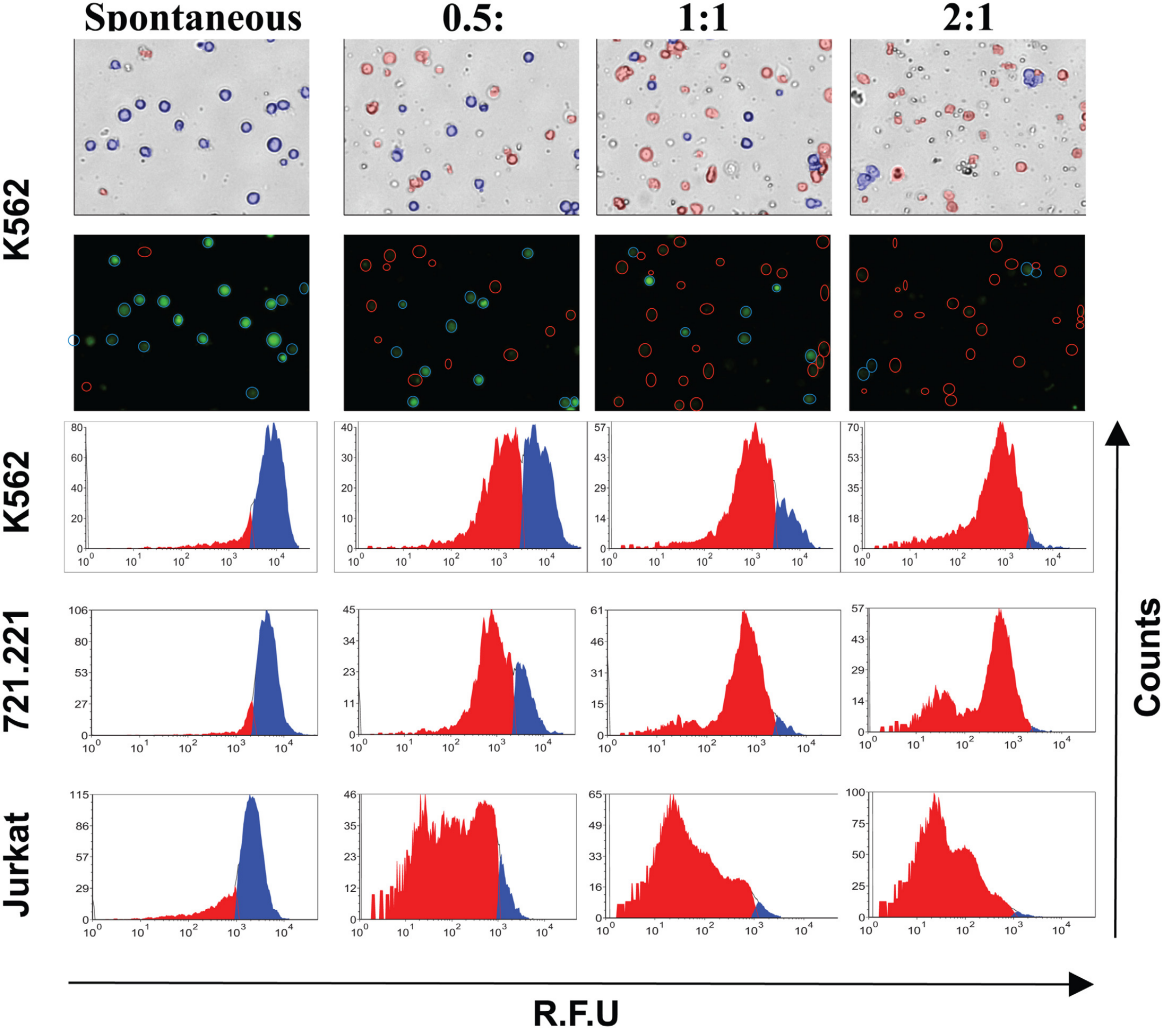
Determination of target cell lysis by image cytometry. The figure shows images from the image cytometer and analysis of fluorescence data using FCS express, to derive live target cell counts. The number of live target cells in the culture was determined by plotting fluorescence intensity of target cells from each E:T ratio compared to spontaneous control. The live K562 cells are shown in blue circles and the lysed cells and apoptotic bodies are highlighted in red circles in the images. Representative fluorescence histograms used for deriving live target cell counts are shown for each tumor cell lines (K562, 721.221 and Jurkat cells).

Next we compared the percent cytotoxicity obtained by calcein release assay to the image cytometer from the same experimental setup. The percent specific lysis from the calcein release assay was calculated from the maximum and spontaneous controls. In the case of image cytometry, in order to derive live cell counts, fluorescence intensity of the target cells was assessed at the various E:T ratios, gating out any object with integrated fluorescence intensity lower than the target cells in the spontaneous control (Fig 3). This fluorescence intensity gating ensures that cells with incomplete calcein release and apoptotic bodies with lower overall fluorescence signal are excluded from the live (brightly fluorescent) cell counts.

**Fig 4:**
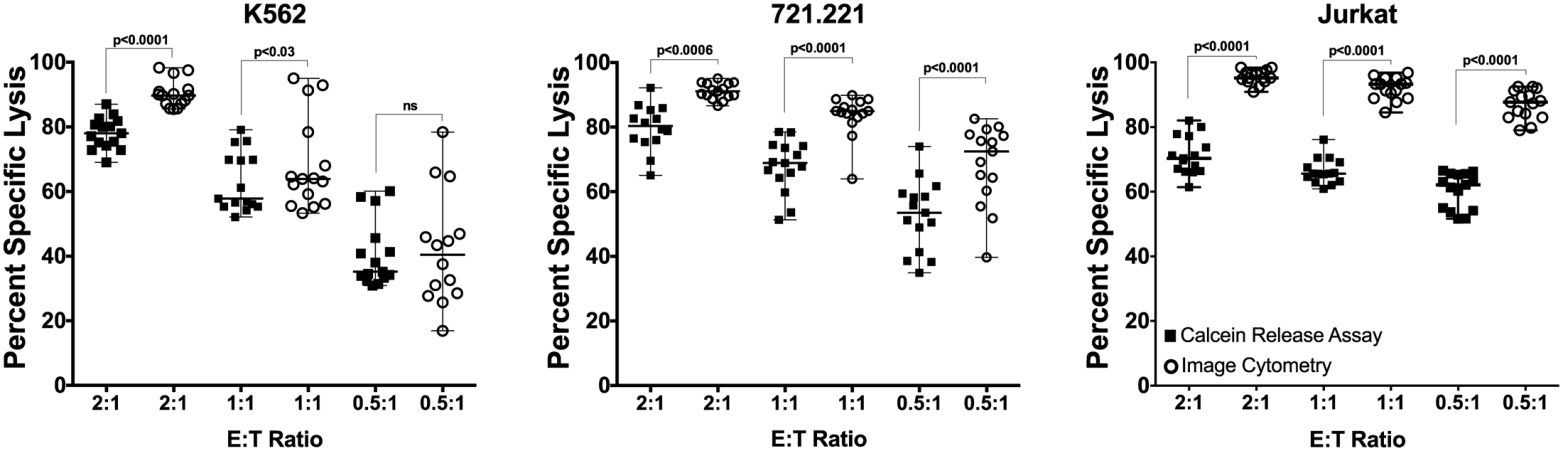
Comparison of percent specific lysis obtained by calcein release assay and image cytometry. Standard calcein release assay and image cytometry were performed within the same assay to determine NK cell killing of K562, 721.221 and Jurkat. The data is shown for each tumor cell line using NK cells expanded from 5 donors (n = 5). Cytotoxicity assay was performed in triplicate for each NK cell donor. Each of the replicate is presented in the plots. The statistical analysis was performed using Wilcoxon matched-pairs signed rank, non-parametric, two-tailed t test and *p* value of <0.05 was considered significant. The data is plotted as median with range.

The percent specific lysis of K562 determined by image cytometry was significantly higher than by the standard calcein release at E:T ratios 2:1 (p < 0.0001) and 1:1 (p < 0.03) however, it was interesting to note that at E:T ratio of 0.5:1 the cytotoxicity was not significantly different between the methods (p = 0.67). In the case of 721.221 cell lines, the percent specific lysis assessed by image cytometry was significantly higher than by the calcein release assay at all E:T ratios (2:1, p = 0.0006; 1:1, p<0.0001; and 0.5:1, p<0.0001). Similarly, the Jurkat cell line showed a significantly higher lysis by image cytometry at all E:T ratios (p<0.0001) (Fig 4). All statistical analyses were done using non-parametric Wilcoxon matched-pair signed rank two-tailed ‘t’ test.

**Fig 5:**
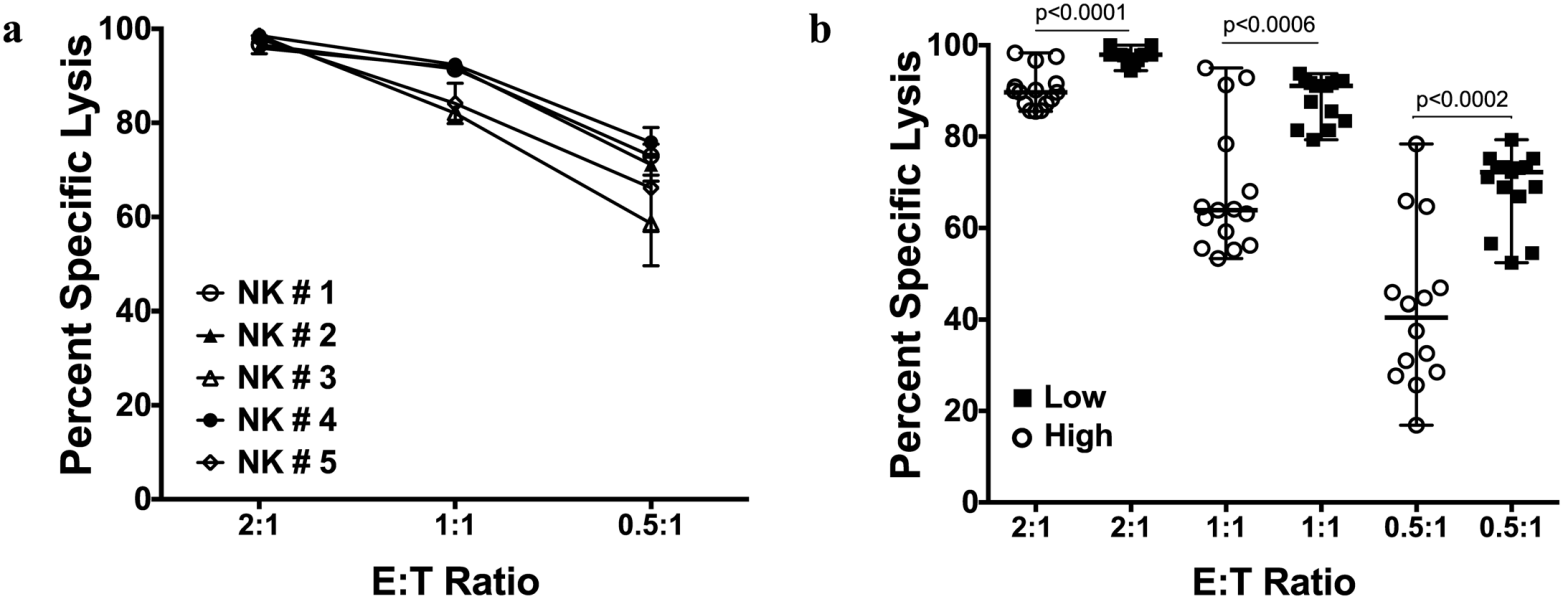
Percent specific lysis of K562 cell line using protocol optimized for direct image cytometry assay. (a) Cytotoxicity against K562 was repeated by image cytometry with reduced target cell number (50,000 cells/well), in a final media volume of 100 μl. After the standard 4-hour incubation the cells were directly resuspended in the 100 μl volume and read using image cytometer. (b) The data shows that with reduced target cell density (Low) the cytotoxicity of NK cells against K562 was significantly higher compared to previous assay with higher target cell density (High) of 100,000 cells/well. The statistical analysis was performed using Wilcoxon matched-pairs signed rank, non-parametric, two-tailed t test and *p* value of <0.05 was considered significant. The data is plotted as median with range.

To make the protocol amenable for smaller sample sizes, we assessed performance of the image cytometry method with reduced numbers of target and effector cells. We performed this assay with expanded NK cells derived from the same 5 donors against the K562 target cell line. For this experiment we seeded 50,000 target cells/well and added NK cells at E:T ratios of 2:1, 1:1 and 0.5:1 in a final volume of 100 μl/well. Three replicates of spontaneous control were included. At the end of a 4-hour incubation, the contents of each well were gently and thoroughly mixed and 20 μl of the cells were recovered and loaded onto disposable slides for imaging. Images were acquired and percent lysis was calculated as before (Fig 5a). It was interesting to note that by reducing the cell density for the assay we observed a significant improvement in percent specific lysis of K562 (Fig 5b) by NK cells from the same donors.

**Fig 6:**
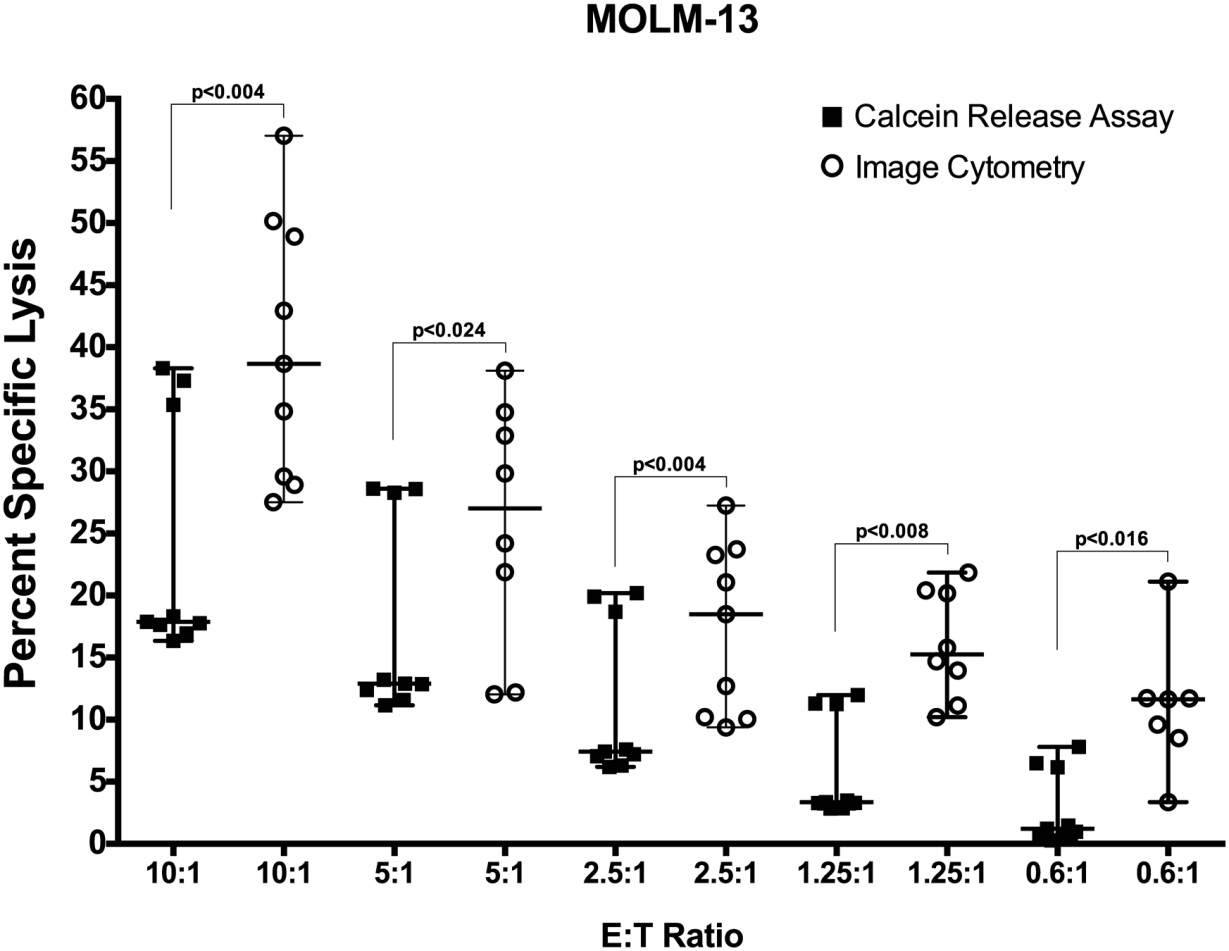
Analysis of percent specific lysis of primary NK cells. The cytotoxicity of primary human NK cells (n = 3) against leukemia cell line MOLM-13 was compared using standard calcein release assay and image cytometry. The image cytometry showed significantly higher lysis of target cells at all E:T ratios tested. The data is shown as median with range. The statistical analysis was performed using Wilcoxon matched-pair signed rank, non-parametric two-tailed *t* test, and *p* value of <0.05 was considered significant.

Finally, we tested the performance of image cytometry for assaying the cytotoxicity of primary NK cells compared to the standard calcein release assay. Since the primary NK cells have lower cytolytic potential than activated and expanded NK cells, we increase the E:T ratios to 10:1. In order to facilitate the increase in E:T ratios and prevent overcrowding the assay wells, we decreased the tumor cell density to 25,000 cells per well in place of the 1x10^5^ and 0.5x10^5^ used before. At the end of 4-hour incubation, the supernatant was analyzed for released calcein and the cell pellet was resuspended and imaged for live fluorescent cells by image cytometer (Fig 6). The percent specific lysis of MOLM-13 cell line by primary NK cells, as shown by image cytometry, was significantly higher across all E:T ratios compared to the standard calcein release assay, demonstrating higher sensitivity and broad applicability of the image cytometry method for assessing NK cell cytotoxicity.

## Discussion

NK cells are cytotoxic lymphocytes and their major function is the direct cytolysis of their target cells. Hence evaluating cytotoxicity is central to studying NK cell function and applications in adoptive immunotherapy.

Numerous methods have been developed for the assessment of immune cell cytotoxicity, the most prominent method being ^51^Chromium release assay (CRA), however, due to concerns over toxicity in handling and disposing radioactive material used in CRA, non-radioactive methods have been reported as alternatives in recent years. We had previously used the calcein release assay to report cytotoxicity of expanded NK cells against various tumor targets [24, 25]. The appeal of the calcein release assay is its simplicity, ease to set up and the use of a non-toxic fluorescent dye. However, we have shown here that the dynamic range of the calcein release assay is divergent for different cell lines, which in turn could affect the accuracy of the assay for some tumor lines. Using live Biostation imaging, we have also shown here that calcein is not released completely from all the target cells lysed during the cytotoxicity assay, and that apoptotic death of target cells during the assay results in the generation of apoptotic bodies that retain calcein without releasing into the culture supernatant for the duration of the standard 4-hour assay. Incomplete calcein release and retention of calcein in apoptotic bodies would result in underestimation of percent lysis by the standard calcein release assay—limitations that could perhaps be shared by most release based assays [18, 31–33].

In this study we report the development of a novel cytotoxicity assay using the Cellometer Vision Image Cytometer and compared its performance to the standard Calcein release assay. We have demonstrated here that image cytometry shows a significantly higher percent specific lysis of target cells compared to calcein release assay within the same experimental setup. The overall improvement in assessment of cytotoxicity could be attributed to the direct imaging of live cells by image cytometry that overcomes the limitations intrinsic to the calcein release assay such as incomplete release of entrapped calcein or retention of calcein within apoptotic bodies after target cell lysis. Using image cytometry, cells with incomplete release of calcein can be excluded from the live cell counts due to their lower fluorescence intensity per cell compared to the spontaneous control, and apoptotic bodies can be excluded from live cell counts due to their overall lower integrated fluorescence intensity per “cell” due to their smaller size compared to intact live cells in the spontaneous control. These results corroborate with other reports that show similar improvement in assessing percent lysis using direct methods compared to CRA—a release-based method [13, 21, 34]. The exception to this observation was found for K562 at E:T ratio of 0.5:1, where the cytotoxicity was not significantly different between these methods. This could possibly be due to the higher dynamic range observed in K562 for the calcein release assay, which could impact the performance of the assay at the lower target cell lysis. This observation nonetheless corroborates with a previous report showing comparable cytotoxicity of K562 between CRA and a flow cytometry-based assay [22]. It is also worth noting that the NK cell cytotoxicity as detected by image cytometry improved when the total cells used in the assay were reduced. Hence, the reduced cell density used here is ideally suited for the assay and based on this data we recommend the use of 50,000 target cells for a cytotoxicity assay against the K562 cell line using image cytometry. We have also tested the performance of the image cytometry platform using primary NK cells compared to the standard calcein release assay. The data demonstrates that the image cytometry remains more sensitive than calcein release assay at determining percent specific lysis even at the lower tumor cell lysis achieved by primary NK cells. In the current study we have used suspension cell lines as targets; however, this method can be applied to adherent cell lines by including a trypsinization or non-enzymatic cell dissociation step similar to those used in flow cytometry-based assays with adherent cells.

We propose that the improved sensitivity offered by image cytometry and the ability of the assay to exclude apoptotic bodies from live cell counts is of considerable advantage over the standard calcein release assay, especially in studies that use chemotherapy drugs to sensitize tumors to NK cell killing. Studies have shown that chemosensitization of tumor cell lines with drugs such as bortezomib, depsipeptide and doxorubicin result in upregulation of TRAIL receptors and caspase activity, which in turn sensitizes them to NK cell mediated apoptosis [35–38]. Since the resulting increase in apoptotic death will produce significantly more apoptotic bodies, the image cytometry method would accurately assess the increase in NK cell mediated cytotoxicity of the tumor cells following this chemosensitization regimen compared to release-based methods such as the calcein release assay. Although, the primary appeal for using release-based assays such as calcein release assay is their relatively high throughput workflow, these methods compromise accuracy of determining percent specific lysis due to the incomplete release of cellular contents upon target cell lysis.

Recently, numerous multi-parametric flow cytometry-based assays have been developed [13, 39–42], that in addition to assessing percent lysis can assay for degranulation, cytokine production, and receptor expression on NK cells as well as surviving target cells. Additionally, the development of single cell cytotoxicity assay using high-resolution microscopy has enabled investigation of multiple parameters of NK cell cytotoxicity at a single cell level, such as serial killing, percentage of effector cells participating in cytotoxicity and kinetics of target cell killing [43–46]. These multiparametric platforms are excellent for answering specialized research questions, however have limited suitability for routine cytotoxicity assays.

Although the assay setup is identical between flow cytometry and image cytometry based cytotoxicity assays, the determination of cell counts by flow cytometry is an indirect process requiring incorporation of counting beads for most flow cytometers. In contrast, the image cytometer directly counts the cells—about 3000 counts in spontaneous control at the lower assay density of 50,000 target cells. The image cytometer is a familiar platform used for routine cell counts and cell-based assays in many laboratories and combines ease with improved accuracy over the standard calcein release assay. We therefore propose image cytometry as an appealing option for performing routine cytotoxicity assays in research labs as well as in Good Manufacturing Practice (GMP) facilities for performing potency assays for adoptive immunotherapy cell products.

## Supporting Information

S1 Movie Live BioStation imaging of CHP 134 lysis by NK cells. The neuroblastoma cell line CHP 134 loaded with calcein, was seeded in Hi-Q4 (4 chambered) dish, and allowed to adhere. Expanded NK cells were added at 10:1 (E:T) ratio, and the cells were imaged at predetermined locations. The movie clip was cropped from a larger field of view to zoom in on lytic interactions. The duration of the movie is 4 hours. The movie shows necrosis-like death of the target cells upon interaction with NK cells and this results in immediate loss of entrapped calcein from the cells. Note that in some instances the entrapped calcein is incompletely lost from the cells.
(MP4)

S2 Movie Live BioStation imaging K562 lysis by NK cells. The K562 cells loaded with calcein were incubated with NK cells at 10:1 (E:T) ratio in Hi-Q4 dish. The movie clip was cropped from a larger field of view to zoom in on lytic interactions. The duration of the movie is 4 hours. The movie shows apoptotic death leading to apoptotic body formation upon lysis by NK cells. These apoptotic bodies retain all the calcein loaded in that particular cell and do not release the entrapped calcein over the 4-hour period.
(MP4)
